# The Co-Chaperone Hch1 Regulates Hsp90 Function Differently than Its Homologue Aha1 and Confers Sensitivity to Yeast to the Hsp90 Inhibitor NVP-AUY922

**DOI:** 10.1371/journal.pone.0049322

**Published:** 2012-11-14

**Authors:** Heather Armstrong, Annemarie Wolmarans, Rebecca Mercier, BaoChan Mai, Paul LaPointe

**Affiliations:** Department of Cell Biology, Faculty of Medicine and Dentistry, University of Alberta, Edmonton, Alberta, Canada; University of Pittsburgh, United States of America

## Abstract

Hsp90 is a dimeric ATPase responsible for the activation or maturation of a specific set of substrate proteins termed ‘clients’. This molecular chaperone acts in the context of a structurally dynamic and highly regulated cycle involving ATP, co-chaperone proteins and clients. Co-chaperone proteins regulate conformational transitions that may be impaired in mutant forms of Hsp90. We report here that the *in vivo* impairment of commonly studied Hsp90 variants harbouring the G313S or A587T mutation are exacerbated by the co-chaperone Hch1p. Deletion of *HCH1*, but not *AHA1*, mitigates the *temperature sensitive* phenotype and high sensitivity to Hsp90 inhibitor drugs observed in *Saccharomyces cerevisiae* that express either of these two Hsp90 variants. Moreover, the deletion of *HCH1* results in high resistance to Hsp90 inhibitors in yeast that express wildtype Hsp90. Conversely, the overexpression of Hch1p greatly increases sensitivity to Hsp90 inhibition in yeast expressing wildtype Hsp90. We conclude that despite the similarity between these two co-chaperones, Hch1p and Aha1p regulate Hsp90 function in distinct ways and likely independent of their roles as ATPase stimulators. We further conclude that Hch1p plays a critical role in regulating Hsp90 inhibitor drug sensitivity in yeast.

## Introduction

The heat shock protein 90 (Hsp90) is a dimeric molecular chaperone responsible for the conformational maturation of specific substrates called ‘client’ proteins [1]. These clients include steroid hormone receptors, kinases and ion channels [2], [3], [4], [5], [6], [7], [8], [9]. Hsp90 is highly conserved from bacteria to humans and is essential in eukaryotes [10], [11]. While the precise mechanism by which Hsp90 chaperones its client proteins remains elusive, it is clear that it acts in the context of a complex ATPase cycle which is regulated by a large cohort of co-chaperone proteins [12], [13].

Hsp90 is integrated with the Hsp70 chaperone system through the action of the co-chaperone Sti1p [14]. Sti1p contains three tetratricopeptide repeat (TPR) domains, two of which interact with short peptides located at the C terminus of Hsp90 and Hsp70 [15]. In this way, Sti1p facilitates the transfer of client proteins from Hsp70 to Hsp90 [14]. The Hsp70 system acts on hydrophobic regions of nascent or unfolded proteins while Hsp90 is thought to facilitate more specific conformational transitions linked to activation or maturation of client proteins [16]. Sti1p is a strong inhibitor of the Hsp90 ATPase activity by preventing dimerization of the N terminal domains [17]. Presumably triggered by appropriate client engagement with Hsp90, ATP and the co-chaperones Cpr6p and Sba1p bind to Hsp90 and synergistically displace Sti1p from Hsp90. At this stage of the Hsp90 cycle, Sba1p interacts with the *N*-terminal ATPase domain of Hsp90, stabilizes ATP binding and slows ATP hydrolysis [18], [19], [20], [21]. The Hsp90 cycle is thought to terminate when ATP hydrolysis occurs, the client protein is released in an activated or mature form, and co-chaperone proteins dissociate. The co-chaperone Aha1p is a potent stimulator of the weak Hsp90 ATPase activity and is thought to play a key role in regulating the kinetics of the Hsp90 cycle [8], [21], [22], [23], [24]. This simplified model does not take into account the numerous other co-chaperone proteins that are known to regulate Hsp90 or introduce specific clients into the cycle, as well as the influence of posttranslational modifications on the Hsp90 system [25], [26], [27], [28], [29]. Consequently, much remains to be elucidated about the nature of the Hsp90 cycle and how it acts to chaperone client proteins.

**Figure 1:**
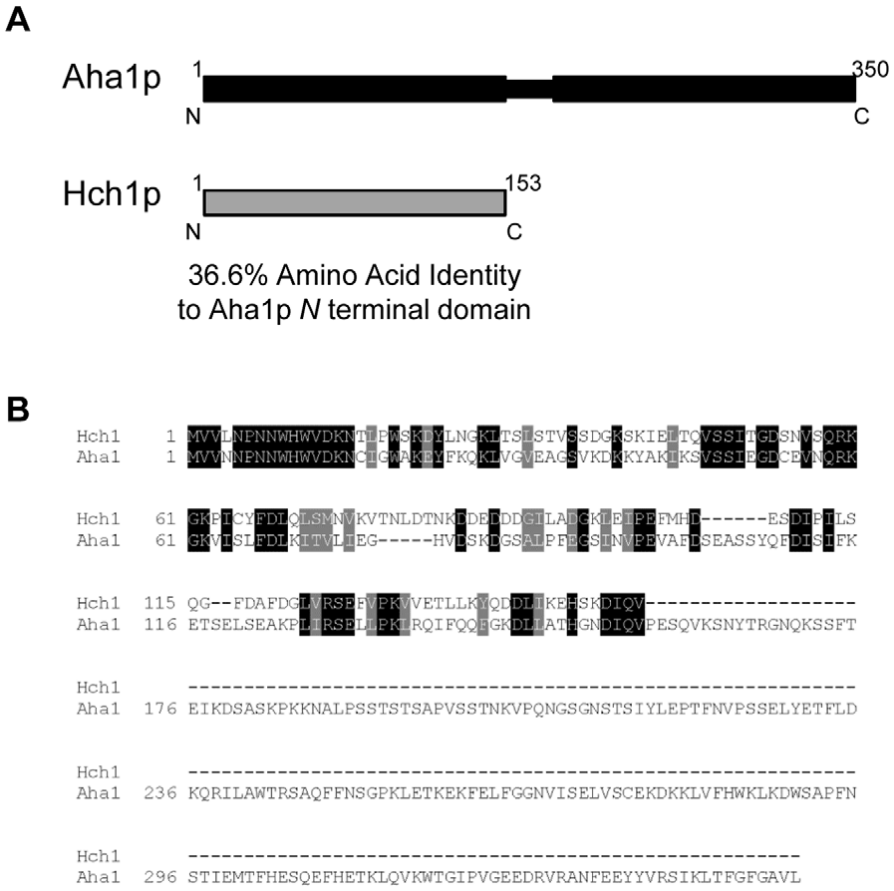
Structure and sequence of Aha1p and Hch1p. **A. Domain structure of the 350 amino acid Aha1p and the 153 amino acid Hch1p.** B. Alignment of Aha1p and Hch1p.

Despite the complexity of the Hsp90 functional cycle, the importance of ATP binding and hydrolysis is well established as Hsp90 mutants that do not bind ATP, or bind ATP but cannot hydrolyze it, do not support viability when expressed as the sole source of Hsp90 in yeast [30], [31]. The importance of ATPase activity for Hsp90 function makes co-chaperones that influence this activity very important to study. Co-chaperones such as Sti1p and Sba1p are known to inhibit the intrinsically low Hsp90 ATPase activity *in vitro* [17], [21], but only the co-chaperone Aha1p has been found to robustly stimulate it [22], [23], [24]. There is a related co-chaperone in yeast called Hch1p that has high sequence identity to the *N*-terminus of Aha1p (36.6% identity, 50.3% similarity; Figure 1) and presumably binds to the same site on the Hsp90 middle domain. Overexpression of Hch1p has been shown to suppress defects in mutant forms of Hsp90 in yeast [32]. However, despite being considered homologues [21], [23], the properties of these two co-chaperone proteins have not been rigorously compared in a yeast system. Both Hch1p and the *N*-terminal domain of Aha1p have been shown to weakly stimulate the Hsp90 ATPase activity but not to the same degree as full-length Aha1p [21]. However, given that the *N*- and *C*-terminal domains of Aha1p bind to Hsp90 at two discrete sites, it is not surprising that full stimulation of Hsp90 requires full length Aha1p (in the middle and *N*- domains of Hsp90 respectively) [24], [33]. Since full-length Aha1p is required for robust stimulation, Hch1p may have a unique function or, alternatively, Hch1p and Aha1p may have a similar function independent of ATPase stimulation.

Yeast is an ideal model system to study Hsp90 function particularly as it relates to co-chaperone regulation of the cycle. Mutants of the inducible yeast Hsp90, Hsp82p, have been generated that appear to have temperature-sensitive (*ts*) defects but their precise molecular impairments are not well understood [34]. Importantly, the different mutant forms of Hsp82p that have been studied have different genetic interactions with the co-chaperone proteins. Genetic interactions have been identified between some of these Hsp82p mutants and certain co-chaperones but these interactions have not been extensively characterized [32], [35], [36]. Of the Hsp82p mutants identified that confer *ts* phenotypes to yeast, only Hsp82p^G170D^ is thought to be thermolabile [34] and biochemical studies have confirmed that several of these Hsp82p mutants do not lose activity at elevated temperatures [22], [37]. However, many Hsp82p mutants that confer *ts* phenotypes to yeast do have altered enzymatic activity under normal conditions (*i.e.* 30°C) suggesting that they are impaired in some biologically relevant conformational transition [37]. Interestingly, the *in vivo* function of one Hsp82p mutant (harbouring the G313S mutation) is strictly dependent on the ordinarily non-essential co-chaperone, Sti1p [38]. Taken together, this suggests that Hsp82p mutants may become aberrantly dependent on specific co-chaperones or antagonized by others. We hypothesized that temperature sensitive growth of yeast expressing mutant forms of Hsp82p would be made worse when *HCH1* or *AHA1* were deleted. These synthetic phenotypes would provide insight into both the molecular defect in the Hsp82p mutant in question and the biological function of Hch1p and Aha1p. To this end, we carried out an analysis of eight different Hsp82p mutants that are associated with *ts* phenotypes in yeast in the context of the co-chaperones Hch1p and Aha1p. Interestingly, we have found that the growth defects in two yeast strains - expressing Hsp82^G313S^ or Hsp82^A587T^ - are rescued when *HCH1*, but not *AHA1*, is deleted. *HCH1* deletion also mitigates the sensitivity to the Hsp90 inhibitor NVP-AUY922 observed in these strains. Our analyses of the phenotypes of strains expressing either of these two mutants as well as of their enzymatic impairments suggest that Hch1p antagonizes Sba1p in manner distinct from Aha1p. We conclude that despite their sequence similarity, Hch1p and Aha1p have distinct roles in the Hsp90 functional cycle that are not linked to the ability to stimulate the Hsp90 ATPase activity.

## Materials and Methods

### Yeast strains/Plasmids

Bacterial expression vectors were constructed from pET11dHis. The *HSP82*, *HCH1* and, *SBA1*, coding sequences were amplified by PCR with primers designed to introduce NdeI and BamHI restriction sites at the 5′ and 3′ ends respectively. The *AHA1* and, *STI1* coding sequences were amplified by PCR with primers designed to introduce NdeI and NotI restriction sites at the 5′ and 3′ ends respectively. These PCR products were digested with NdeI and BamHI or NotI for ligation into similarly cut pET11dHis. Proteins harbouring tandem N-terminal 6xHis and myc tags were expressed from a derivative of pET11dHis where the coding sequence for the myc epitope was fused in-frame with the 6xHis-tag sequence and upstream of the NdeI site. Co-chaperone coding sequences were cloned into this pET11dHismyc vector as described above. The G313S and A587T mutations were introduced into the *HSP82* coding sequence using Quikchange™ mutagenesis according to the manufacturers protocol (Agilent).

We constructed our p404TDH3 yeast integrating vectors by cloning the SacI-KpnI fragment from pRS426TDH3 [39] into similarly digested pRS404. We then amplified the *HCH1* coding sequence by PCR with primers designed to introduce a BglII site, 6xHis-tag, and NdeI site at the 5′ end and a XhoI site and nested BamHI site at the 3′ end. This PCR product was digested with BglII and XhoI and then cloned into pRS404TDH3 that was digested with BamHI and XhoI. The resultant plasmid, p404TDH3HisHCH1, was used as an acceptor plasmid that *HSP82* (wildtype and mutant) sequences could be subcloned into from our pET11dHis vectors (digested with NdeI and BamHI).

We constructed our p41KanTEF vector by digesting the TEF2 promoter and CYC1 terminator fragment of p414TEF [39] with SacI and KpnI, and ligating into similarly cut pRS41K [40]. The *HCH1* and *AHA1* coding sequences were amplified by PCR with primers designed to introduce a BamHI site at the 5′ end and a myc tag and XhoI site at the 3′ end. These PCR products were then digested with BamHI and XhoI and cloned into similarly cut p41Kan to yield p41KanTEFHch1myc and p41KanTEFAha1myc.

All yeast strains used in this study (Table 1) were derived from ΔPCLDa [34]. Briefly, we knocked out the *HCH1* and *AHA1* coding sequences with the *HIS3* selectable marker using PCR-mediated gene replacement. Histidine autotrophs were selected on SC-histidine plates and clones were analyzed by PCR to verify gene replacement. We then integrated pRS404-derived vectors designed to constitutively express *N*-terminally His-tagged Hsp82p from the TDH3 promoter. p404TDH3HisHSP82, p404TDH3HisHSP82-G313S, and p404TDH3HisHSP82-A587T, were linearized with HindIII and transformed into yeast (either ΔPCLDa, ΔPCLDa*Δhch1*, or ΔPCLDa*Δaha1*) by the lithium acetate method [41]. Transformants were selected on SC–tryptophan and shuffling of the URA3-marked episome harbouring wildtype *HSC82* was carried out with 5-fluoroorotic acid [42]. In the case of yeast strains transformed with our p41KanTEF plasmids, transformants were selected on YPD supplemented with 200 mg/L G418 (HyClone, Utah, USA).

**Table 1:** Yeast strains used in this study.

Yeast Strain	Genotype	Source
ΔPCLD82a		Nathan and Lindquist (1995)
ip82a	*MATa ade2-1 leu2-3,112 his3-11,15 trp1-1 ura3-1 can1-100 hsc82::LEU2 hsp82::LEU2 pTTDH3-HSP82*	Nathan and Lindquist (1995)
ip82a *hch1*	*MATa ade2-1 leu2-3,112 his3-11,15 trp1-1 ura3-1 can1-100 hch1::URA3 hsc82::LEU2 hsp82::LEU2 pTTDH3-HSP82*	This study
ip82a *aha1*	*MATa ade2-1 leu2-3,112 his3-11,15 trp1-1 ura3-1 can1-100 aha1::URA3 hsc82::LEU2 hsp82::LEU2 pTTDH3-HSP82*	This study
ip82a *hch1 aha1*	*MATa ade2-1 leu2-3,112 his3-11,15 trp1-1 ura3-1 can1-100 hch1::HIS3 aha1::URA3 hsc82::LEU2 hsp82::LEU2 pTTDH3-HSP82*	This study
ipG313Sa	*MATa ade2-1 leu2-3,112 his3-11,15 trp1-1 ura3-1 can1-100 hsc82::LEU2 hsp82::LEU2 pTTDH3-HSP82^G313S^*	Nathan and Lindquist (1995)
ipG313Sa *hch1*	*MATa ade2-1 leu2-3,112 his3-11,15 trp1-1 ura3-1 can1-100 hch1::URA3 hsc82::LEU2 hsp82::LEU2 pTTDH3-HSP82^G313S^*	This study
ipG313Sa *aha1*	*MATa ade2-1 leu2-3,112 his3-11,15 trp1-1 ura3-1 can1-100 aha1::URA3 hsc82::LEU2 hsp82::LEU2 pTTDH3-HSP82^G313S^*	This study
ipG313Sa *hch1 aha1*	*MATa ade2-1 leu2-3,112 his3-11,15 trp1-1 ura3-1 can1-100 hch1::HIS3 aha1::URA3 hsc82::LEU2 hsp82::LEU2 pTTDH3-HSP82^G313S^*	This study
ipA587Ta	*MATa ade2-1 leu2-3,112 his3-11,15 trp1-1 ura3-1 can1-100 hsc82::LEU2 hsp82::LEU2 pTTDH3-HSP82^A587T^*	Nathan and Lindquist (1995)
ipA587Ta *hch1*	*MATa ade2-1 leu2-3,112 his3-11,15 trp1-1 ura3-1 can1-100 hch1::URA3 hsc82::LEU2 hsp82::LEU2 pTTDH3-HSP82^A587T^*	This study
ipA587Ta *aha1*	*MATa ade2-1 leu2-3,112 his3-11,15 trp1-1 ura3-1 can1-100 aha1::URA3 hsc82::LEU2 hsp82::LEU2 pTTDH3-HSP82^A587T^*	This study
ipA587Ta *hch1 aha1*	*MATa ade2-1 leu2-3,112 his3-11,15 trp1-1 ura3-1 can1-100 hch1::HIS3 aha1::URA3 hsc82::LEU2 hsp82::LEU2 pTTDH3-HSP82^A587T^*	This study
ipT22Ia	*MATa ade2-1 leu2-3,112 his3-11,15 trp1-1 ura3-1 can1-100 hsc82::LEU2 hsp82::LEU2 pTTDH3-HSP82^T22I^*	Nathan and Lindquist (1995)
ipA41Va	*MATa ade2-1 leu2-3,112 his3-11,15 trp1-1 ura3-1 can1-100 hsc82::LEU2 hsp82::LEU2 pTTDH3-HSP82^A41V^*	Nathan and Lindquist (1995)
ipG81Sa	*MATa ade2-1 leu2-3,112 his3-11,15 trp1-1 ura3-1 can1-100 hsc82::LEU2 hsp82::LEU2 pTTDH3-HSP82^G81S^*	Nathan and Lindquist (1995)
ipT101Ia	*MATa ade2-1 leu2-3,112 his3-11,15 trp1-1 ura3-1 can1-100 hsc82::LEU2 hsp82::LEU2 pTTDH3-HSP82^T101I^*	Nathan and Lindquist (1995)
ipG170Da	*MATa ade2-1 leu2-3,112 his3-11,15 trp1-1 ura3-1 can1-100 hsc82::LEU2 hsp82::LEU2 pTTDH3-HSP82^G170D^*	Nathan and Lindquist (1995)
ipE381Ka	*MATa ade2-1 leu2-3,112 his3-11,15 trp1-1 ura3-1 can1-100 hsc82::LEU2 hsp82::LEU2 pTTDH3-HSP82^E381K^*	Nathan and Lindquist (1995)
iT HisHsp82	*MATa ade2-1 leu2-3,112 his3-11,15 trp1-1 ura3-1 can1-100 hsc82::LEU2 hsp82::LEU2 p404TDH3-HisHSP82*	This study
iT HisHsp82 *hch1*	*MATa ade2-1 leu2-3,112 his3-11,15 trp1-1 ura3-1 can1-100 hch1::HIS3 hsc82::LEU2 hsp82::LEU2 p404TDH3-HisHSP82*	This study
iT HisHsp82 *aha1*	*MATa ade2-1 leu2-3,112 his3-11,15 trp1-1 ura3-1 can1-100 aha1::HIS3 hsc82::LEU2 hsp82::LEU2 p404TDH3-HisHSP82*	This study
iT HisG313S	*MATa ade2-1 leu2-3,112 his3-11,15 trp1-1 ura3-1 can1-100 hsc82::LEU2 hsp82::LEU2 p404TDH3-HisHSP82^G313S^*	This study
iT HisG313S *hch1*	*MATa ade2-1 leu2-3,112 his3-11,15 trp1-1 ura3-1 can1-100 hch1::HIS3 hsc82::LEU2 hsp82::LEU2 p404TDH3-HisHSP82^G313S^*	This study
iT HisG313S *aha1*	*MATa ade2-1 leu2-3,112 his3-11,15 trp1-1 ura3-1 can1-100 aha1::HIS3 hsc82::LEU2 hsp82::LEU2 p404TDH3-HisHSP82^G313S^*	This study
iT HisA587T	*MATa ade2-1 leu2-3,112 his3-11,15 trp1-1 ura3-1 can1-100 hsc82::LEU2 hsp82::LEU2 p404TDH3-HisHSP82^A587T^*	This study
iT HisA587T *hch1*	*MATa ade2-1 leu2-3,112 his3-11,15 trp1-1 ura3-1 can1-100 hch1::HIS3 hsc82::LEU2 hsp82::LEU2 p404TDH3-HisHSP82^A587T^*	This study
iT HisA587T *aha1*	*MATa ade2-1 leu2-3,112 his3-11,15 trp1-1 ura3-1 can1-100 aha1::HIS3 hsc82::LEU2 hsp82::LEU2 p404TDH3-HisHSP82^A587T^*	This study

### Growth assays

Strains were grown in defined media or YPD (where indicated), diluted to 1×10^8^ cells per mL and 10-fold serial dilutions were prepared as indicated. Five µL drops were placed on agar plates (YPD or defined, with or without NVP-AUY922 where indicated) and grown for 72 hours at the indicated temperatures.

### Protein Expression and Purification

*S. cerevisiae* Hsp82p, Hsp82p^G313S^, Hsp82p^A587T^, Aha1p, Hch1p, Sti1p and Sba1p were expressed in *Escherichia coli* strain BL21(DE3) from pET11dHis (Stratagene, La Jolla, California, USA). Cells were grown to an OD_600_ of 0.8, induce with 1 mM IPTG and grown overnight at 37°C. Cells were harvested by centrifugation, resuspended in lysis buffer (25 mM NaH_2_PO_4_ pH 7.2, 500 mM NaCl, 1 mM MgCl_2_, 20 mM Imidazole, 5 mM β-mercaptoethanol) and lysed using an Avestin Emulsiflex C3 (Avestin, Ottawa, Ontario, Canada). Lysates were clarified by ultracentrifugation and His-tagged proteins were isolated on a HisTrap FF column using an AKTA Explorer FPLC (GE Healthcare). Isolated 6xHis-tagged proteins were then concentrated and further purified by size exclusion chromatography on a Superose6 (Hsp82p and variants) or Superdex75 (Aha1p, Hch1p, Sba1p and Sti1p) column (GE Healthcare).

### ATPase Assays

ATPase assays were carried out using the enzyme-coupled assay as previously described [31]. All reactions were carried out in 100 µl volumes in a 96-well plate and in triplicate. Average values of those triplicates are shown with error expressed as standard error of the mean. The final conditions in all of our reactions (except protein components which are specified in each experiment) are 25 mM Hepes pH 7.2, between 1 and 22 mM NaCl (depending on proteins added), 5 mM MgCl_2_, 1 mM DTT, 0.3 mM NADH, 2 mM ATP, 1 mM phosphoenol pyruvate, and 2.5 µL of pyruvate kinase/lactate dehydrogenase (Sigma) in 100 µL.

### Lysate Generation and Western Blotting

Yeast were grown overnight at 30°C in appropriate media. 0.5 OD_600_ units of cells were transferred to a microfuge tube, washed with distilled water and then pelleted for processing. To each cell pellet, 90 µL of lysis buffer (2.2 M NaOH, 1 mM β-mercaptoethanol, 10 mM PMSF) was added and samples vortexed twice for 1 minute. 250 µL of ice cold 100% TCA was added and samples were vortexed briefly and then precipitated protein was pelleted in a cold microcentrifuge. Pellets were washed twice in ice cold acetone, dried and then resuspended in sample buffer for analysis by SDS-PAGE and western blotting. Myc-tagged proteins were detected with mouse anti-myc monoclonal antibody [43] (4A6 Millipore) and His-tagged proteins were detected with rabbit anti-His tag antibody (#2365, Cell Signaling Technology). Anti-Sba1p antibodies were kindly provided by Dr. Brian Freeman (University of Illinois at Urbana Champaign). Anti-actin antibodies were kindly provided by Dr. Gary Eitzen (University Alberta). Anti-Aha1p and anti-Sti1p antibodies were raised in rabbits against the peptides CESQVKSNYTRGNQK and CDINQSNSMPKEPET respectively (Genscript).

For 6xHis-tag pulldown experiments yeast were grown in 250 mL cultures of YPD at 30°C overnight. Cultures were harvested by centrifugation at 3500× g for 15 minutes at 4°C. Cells were washed twice with 25 mL of water and then stored at −20°C for later processing. Frozen pellets were thawed and resuspended in lysis buffer (50 mM Tris pH 7.5, 100 mM KCl, 5 mM MgCl_2_, 20 mM Na_2_MoO_4_, 20% Glycerol, 5 mM β-mercaptoethanol, HALT EDTA-free protease inhibitor (Thermo scientific). Cells were lysed by continuous passage through an Emulsiflex C3 (Avestin, Ottawa, Ontario, Canada) for 3 minutes. Lysates were clarified by centrifugation at 41,000× g for 10 minutes at 4°C. 1.9 mL of lysate was transferred to a 2 mL screw-cap tube with 50 µL of a 50∶50 slurry of Ni-NTA beads (Qiagen). Samples were incubated on a rotator overnight at 4°C. The following day, beads were pelleted by centrifugation for 5 minutes at 1000 rpm at 4°C and the beads were washed twice with 500 µL lysis buffer supplemented with 35 mM imidazole and 0.1% Tween-20. Bound complexes were eluted in 250 µL elution buffer (25 mM NaH_2_PO_4_ pH 7.2, 500 mM NaCl, 1 mM MgCl_2_, 1M imidazole, 5 mM β-mercaptoethanol). Eluted proteins were analyzed by SDS-PAGE followed by coomassie staining and western blotting.

### In vitro assembly and analysis of Hsp82p complexes with co-chaperones

Purified Hsp82p (wildtype or mutant) was mixed with indicated co-chaperone proteins in 50 µL volumes (final buffer condition 25 mM Hepes pH 7.2, 10 mM NaCl, 5 mM MgCl_2_, 0.1% Tween-20). These reactions were supplemented with 1 mM ADP or AMPPnP, and 10 µL of Ultralink Protein G beads that had been coupled to anti-myc monoclonal antibodies at a concentration of 5 µg antibody per 1 µl of beads. These reactions were incubated on a rotator at room temperature for 90 minutes. Beads were pelleted, washed once in 250 µl of binding buffer and then run on SDS-PAGE.

## Results

### Deletion of HCH1 rescues growth of yeast expressing Hsp82p^G313S^ or Hsp82p^A587T^

The multitudes of processes that Hsp90 regulates are thought to depend on a common core cycle of client interaction, ATP binding, and then release of the mature client coincident with ATP hydrolysis [44]. Interestingly, direct measurements of Hsp90 conformations in solution have shown that there are indeed multiple conformations that Hsp90 can adopt but that the acquisition of these conformational states is highly stochastic, and not obligately ordered regardless of the presence or absence of nucleotide [45]. Certainly, the fact that the ability to bind and hydrolyze ATP is an absolute requirement for Hsp90 function *in vivo*, and that most co-chaperones are not essential for yeast viability suggest that Hsp90 can complete its conformational cycle and act on client proteins largely on its own. However, co-chaperones can become essential when mutant forms of Hsp82p are expressed as the sole source of Hsp90 in the cell [38]. Growth defects in yeast that express some mutated forms of Hsp82p have also been shown to be rescued by co-chaperone overexpression [34], [46]. Consequently, mutant forms of Hsp82p can be valuable tools in understanding co-chaperone function.

**Figure 2:**
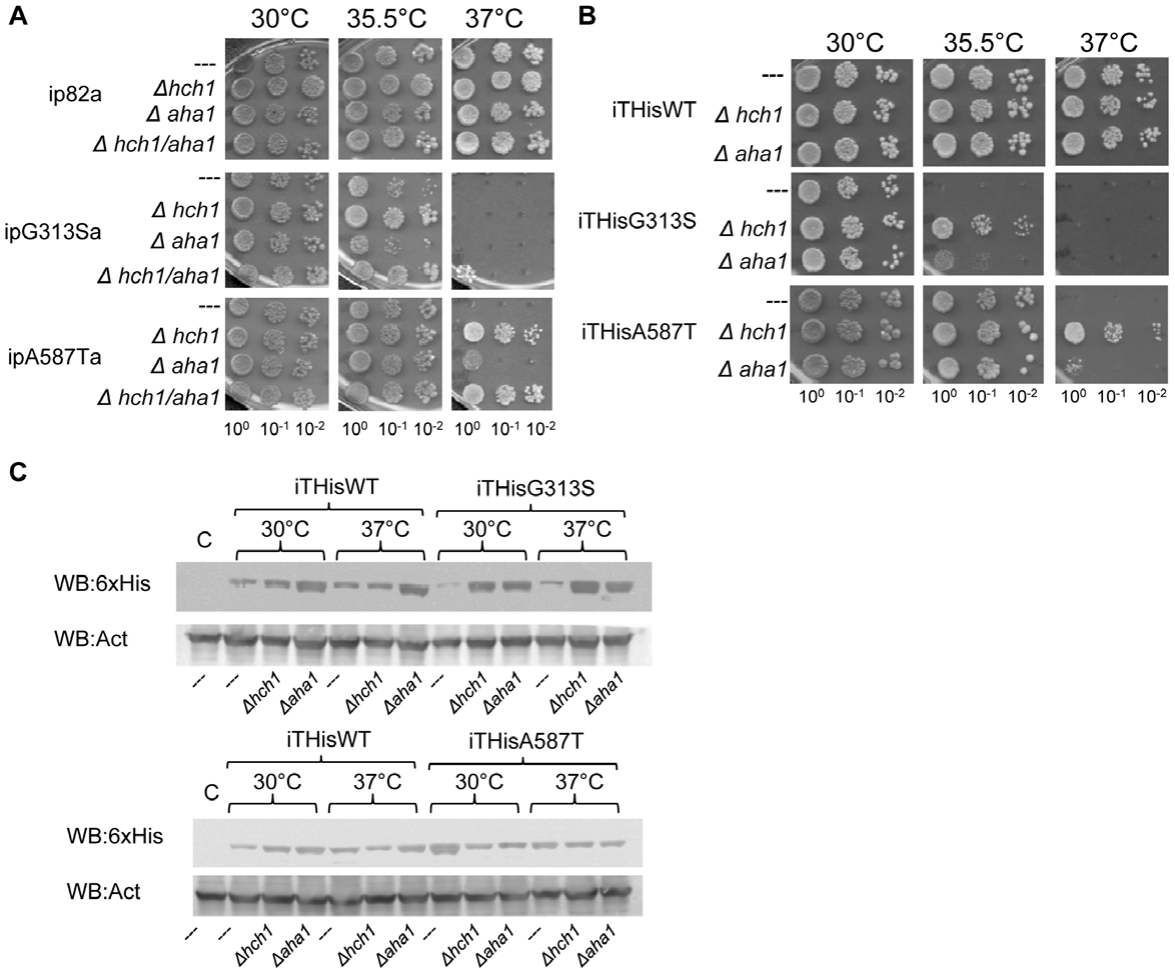
Deletion of *HCH1*, but not *AHA1*, alleviates temperature sensitivity in *S. cerevisiae* expressing Hsp82p^G313S^ or Hsp82p^A587T^ as the sole source of Hsp90. Cells were grown overnight in YPD at 30°C and then diluted to 1×10^8^ cells per mL. 10-fold serial dilutions were prepared and 5 µL aliquots were spotted on YPD-agar plates. A. Viability of mutant strains (expressing untagged Hsp82p) grown at 30, 35.5 and 37°C on YPD agar plates; B. Viability of mutant strains (expressing His-tagged Hsp82p) grown at 30, 35.5 and 37°C on YPD agar plates. C. Western analysis of HisHsp82p expression versus actin in mutant strains.

**Figure 3:**
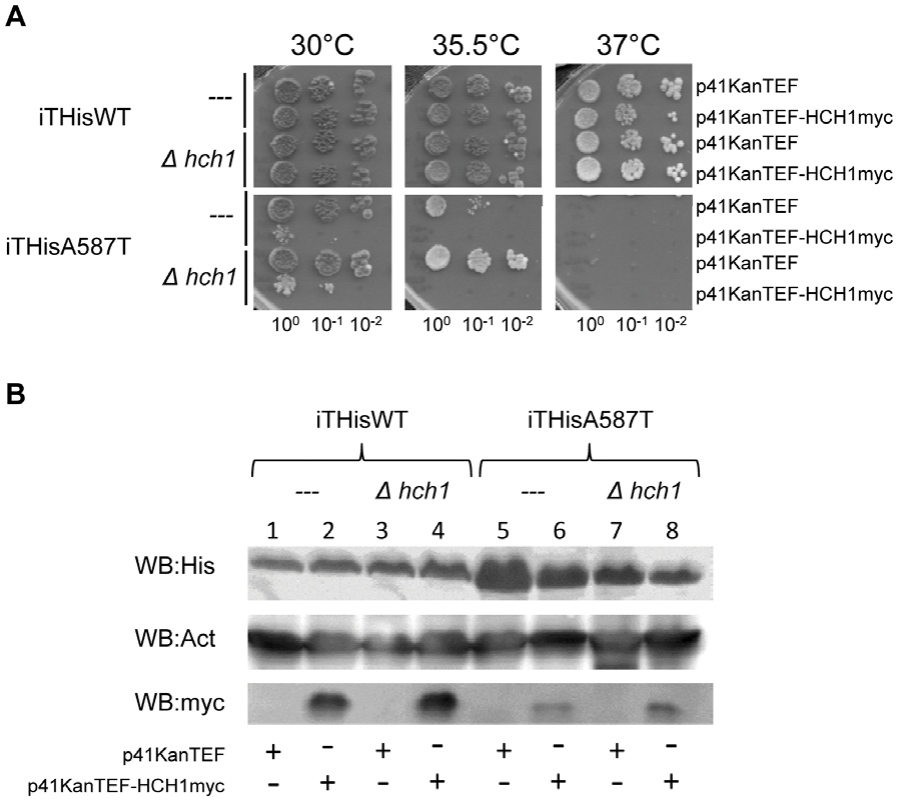
Overexpression of C-terminally myc-tagged Hch1p impairs growth of yeast expressing Hsp82p^A587T^ but not yeast expressing wildtype Hsp82p. A. Cells were grown overnight in YPD supplemented with 200 mg/L G418 and then diluted to 1×10^8^ cells per mL. 10-fold serial dilutions were prepared and 5 µL aliquots were spotted on YPD agar plates supplemented with 200 mg/L G418 and grown for 2 days at 30, 35.5 and 37°C. B. Western analysis of yeast shown in 3A with anti-myc tag, anti-His tag and anti-actin antibodies. Levels of mycHch1p (lanes 2, 4, 6, and 8) in strains expressing Hsp82p (lanes 1–4), or Hsp82p^A587T^ (lanes 5–8) with (lanes 3,4, 7, 8) or without (lanes 1, 2, 5, 6) *HCH1* deletion. (Anti-myc antibody 9E10).

We hypothesized that specific Hsp82p mutants would have synthetic phenotypes related to *HCH1* or *AHA1* deletion. To test this, we deleted either *HCH1* or *AHA1* in each of eight *ts* strains (ip82a, ipT22Ia, ipA41Va, ipG81Sa, ipT101Ia, ipG170Da, ipG313Sa, ipE381Ka, ipA587Ta [34]) and assessed their growth at a range of temperatures (Table 2). Surprisingly, of the eight strains we examined, two of these yeast strains – ipG313Sa and ipA587Ta – were rescued when *HCH1*, but not *AHA1*, was deleted (Figure 2A). To confirm these results, we generated our own strains derived from the original plasmid shuffling strain, ΔPCLD82a (see materials and methods). These strains expressed His-tagged Hsp82p harbouring the same point mutations and showed the same phenotype in plate dilution assays (Figure 2B). To rule out the possibility that *HCH1* deletion resulted in increased stability or expression of Hsp82p we performed western blot analysis of lysates from each of our strains grown for 7 hours at 30°C or the restrictive 37°C. This showed that wildtype Hsp82p and Hsp82p^G313S^ and Hsp82p^A587T^ were expressed at comparable levels relative to actin at both temperatures tested (Figure 2C). However, in yeast expressing His-tagged Hsp82p^G313S^, the deletion of either *HCH1* or *AHA1* resulted in a significant increase in Hsp82p levels. While this does not account for the growth rescue associated with *HCH1* deletion (since *AHA1* deletion had no effect on growth) it is not clear if these differences are due to clonal differences in Hsp82p expression or a direct result of co-chaperone deletion (see next paragraph and Figure 3).

**Table 2:** Effect of *HCH1* or *AHA1* deletion on yeast growth.

Yeast Strain	*Δhch1*	*Δaha1*
ip82a	-	-
ipT22Aa	*ND* *	-
ipA41Va	*-*	-
ipG81Sa	*-*	-
ipT101Ia	*-*	-
ipG170Da	*-*	-
ipG313Sa	↑↑	-
ipE381Ka	*ND* **	-
ipA587Ta	↑↑	-

* - ipT22Ia appeared to have two copies of HCH1 (possibly due to chromosomal rearrangement or duplication) and was not tested.

** - We were unable to obtain a *HCH1* deletion for this strain.

To test the possibility that overexpression of *HCH1* would have the opposite effect in these strains we constructed G418 selectable plasmids that encode *C*-terminally myc-tagged Hch1p under the control of the constitutive TEF2 promoter (p41KanTEF and p41KanTEFHch1myc - see materials and methods). The overexpression Hch1p did not affect the growth of yeast expressing wildtype Hsp82p at 30°C but severely reduced the growth of yeast expressing Hsp82p^A587T^ (Figure 3A). Moreover, the improved growth we observed in strains with *HCH1* deleted was reversed when Hch1p was over-expressed (Figure 3A). Unfortunately, we were unable to get G418-resistant clones when we transformed the Hch1p overexpression plasmid into yeast expressing Hsp82p^G313S^. While this does not prove that overexpression of Hch1p is toxic to this strain it is certainly a strong indication to that effect. We confirmed the overexpression of myc-tagged Hch1p by western blot (Figure 3B) and found that the different levels of HisHsp82 observed in Figure 2C were not altered (compared to actin control) when Hch1p was over-expressed. This suggests that the variable expression of Hsp82p in our strains is likely a result of clonal variation rather than a direct consequence of co-chaperone expression. Regardless, the expression level of HisHsp82p does correlate with the growth phenotypes we observed suggesting that Hch1p expression is responsible for changes in temperature sensitive growth.

### Deletion of HCH1 confers resistance to the Hsp90 inhibitor NVP-AUY922 in vivo

**Figure 4:**
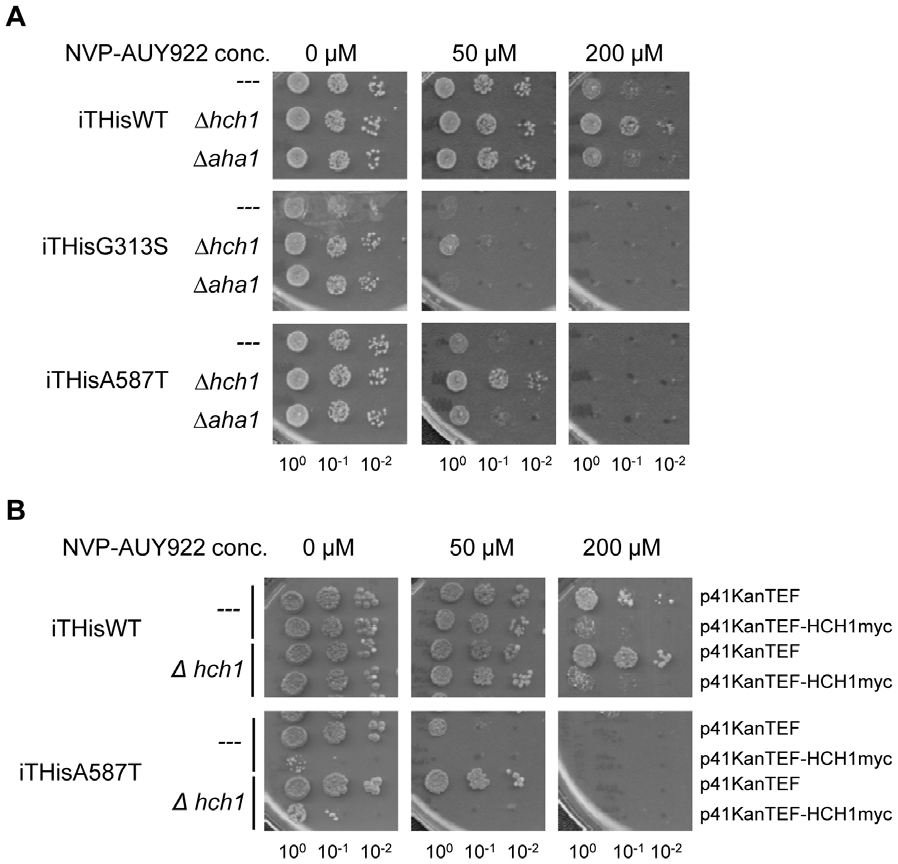
Hch1p regulates Hsp90 inhibitor drug sensitivity in yeast. A. Deletion of *HCH1* confers resistance to Hsp90 inhibitor NVP-AUY922 in yeast expressing Hsp82p, Hsp82p^G313S^ or Hsp82p^A587T^. Cells were grown overnight in YPD and then diluted to 1×10^8^ cells per mL. 10-fold serial dilutions were prepared and 5 µL aliquots were spotted on YPD agar plates supplemented with indicated concentrations of NVP-AUY922. B. Overexpression of myc-tagged Hch1p results in hypersensitivity to Hsp90 inhibitor NVP-AUY922 in yeast. Cells were grown overnight in YPD supplemented with 200 mg/L G418 and then diluted to 1×10^8^ cells per mL. 10-fold serial dilutions were prepared and 5 µL aliquots were spotted on YPD agar plates supplemented with 200 mg/L G418 and indicated concentrations of NVP-AUY922.

Another phenotype attributed to strains expressing Hsp82p^G313S^ or Hsp82p^A587T^ as the sole source of Hsp90 is hyper-sensitivity to Hsp90-inhibiting drugs like geldanamycin [47]. To overcome problems associated with the poor solubility of geldanamycin and its derivatives, we tested our strains for resistance to the relatively new Hsp90 inhibitor NVP-AUY922 [48], [49], [50]. NVP-AUY922 binds to the ATP binding pocket of Hsp90 with very high affinity (very low nanomolar range for human Hsp90) and is highly soluble which makes it ideal for use in our assays [48], [49], [50]. Interestingly, deletion of *HCH1*, but not *AHA1*, increased Hsp90-inhibitor resistance in yeast expressing Hsp82p^G313S^ or Hsp82p^A587T^ (Figure 4A). Moreover, even yeast expressing wildtype Hsp82p became more resistant to NVP-AUY922 when *HCH1* was deleted (Figure 4A). We then transformed our strains with the p41KanTEFHch1myc plasmid to determine if Hch1p overexpression had the opposite effect. As expected, Hch1p overexpression was deleterious to yeast expressing Hsp82p^A587T^ and reversed the NVP-AUY922 resistance conferred by *HCH1* deletion in our strains (Figure 4B). Interestingly, Hch1p overexpression conferred NVP-AUY922 hypersensitivity to yeast expressing wildtype Hsp82p (Figure 4B) suggesting that Hch1p plays a critical role in Hsp90 inhibitor resistance and sensitivity in yeast.

**Figure 5:**
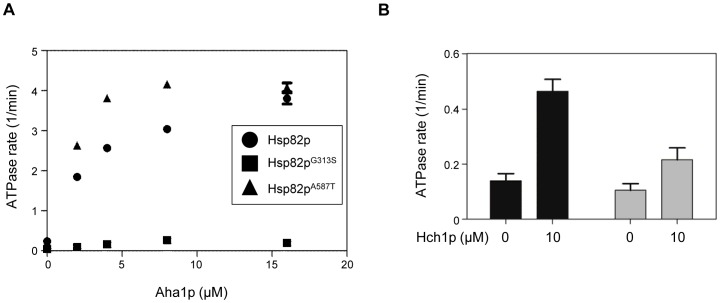
ATPase stimulation of Hsp82p, Hsp82p^G313S^ and Hsp82p^A587T^. A. Stimulation of the ATPase activity of wildtype Hsp82p (circles), Hsp82p^G313S^ (squares), and Hsp82p^A587T^ (triangles) by increasing concentrations of Aha1p. B. Stimulation of the ATPase activity of wildtype Hsp82p (black bars), and Hsp82p^A587T^ (grey bars) by Hch1p. ATPase rate shown in µM ATP hydrolyzed per minute per µM of enzyme (1/min). Reactions contained 2 µM Hsp82p and indicated concentration of Aha1p or Hch1p.

*Characterization of intrinsic, Hch1p-stimulated, and Aha1p-stimulated ATPase activity of Hsp82p mutants -* Hsp82p mutants that confer *ts* growth to yeast do not necessarily have similar defects in their ATPase activity [37]. Owing to the phenotypic similarity of yeast strains expressing Hsp82p^G313S^ or Hsp82p^A587T^ we wondered if the enzymatic properties of these Hsp82p mutants would be similar as well. To this end, we purified recombinant wildtype and mutant forms of Hsp82p from *E. coli* for *in vitro* ATPase testing. We tested wildtype Hsp82p, Hsp82p^G313S^ and Hsp82p^A587T^ for unstimulated, and Aha1p-stimulated ATPase activity. Surprisingly, despite the similarity of phenotypes in yeast expressing either of these two Hsp82p variants, their biochemical properties were very different. Consistent with previous reports, Hsp82p^A587T^ was very similar to wildtype in its unstimulated ATPase rate [51] as well as its Aha1p-stimulated rate (Figure 5A). In contrast, Hsp82p^G313S^ was only weakly stimulated by Aha1p at concentrations up to 16 µM (Figure 5A) and its unstimulated rate was virtually undetectable. When we performed similar experiments with Hch1p we found that Hch1p-stimulated ATPase activity of Hsp82p^A587T^ was much weaker than that of wildtype Hsp82p (Figure 5B) (we did not test Hsp82p^G313S^ owing to its extremely low ATPase activity).

### Inhibition of Stimulated ATPase reactions by Sba1p and Sti1p

**Figure 6:**
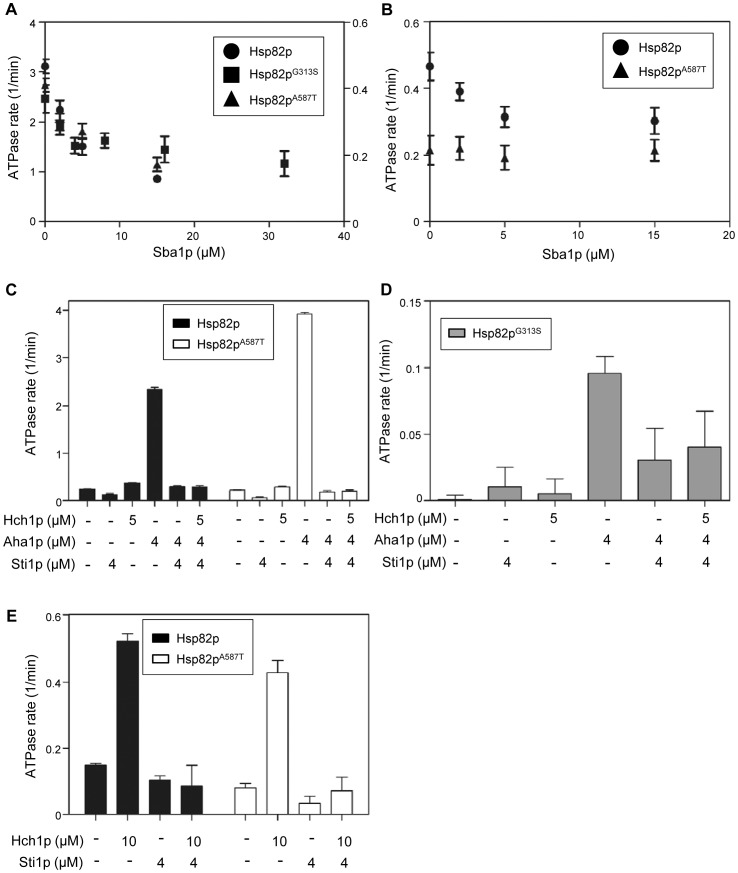
Inhibition of Aha1p-stimulated ATPase activity of Hsp82p, Hsp82p^G313S^ and Hsp82p^A587T^. A. Inhibition of Aha1p-stimulated ATPase activity of wildtype Hsp82p (circles), Hsp82p^G313S^ (squares), and Hsp82p^A587T^ (triangles) by increasing concentrations of Sba1p. ATPase rate for wildtype Hsp82p and Hsp82p^A587T^ shown on left axis (Reactions contained 2 µM Hsp82p, 10 µM Aha1p and indicated concentrations of Sba1p). ATPase rate for Hsp82p^G313S^ shown on right axis (reactions contained 5 µM Hsp82p, 10 µM Aha1p and indicated concentrations of Sba1p). B. Inhibition of Hch1p-stimulated ATPase activity of wildtype Hsp82p (circles), and Hsp82p^A587T^ (triangles) by increasing concentrations of Sba1p. Reactions contained 2 µM Hsp82p, 10 µM Hch1p and indicated concentrations of Sba1p. C. Inhibition of Aha1p-stimulated ATPase activity of wildtype Hsp82p (black bars) and Hsp82p^A587T^ (white bars) by Sti1p. All reactions contained 2 µM Hsp82p and indicated concentrations of co-chaperones. D. Inhibition of Aha1p-stimulated ATPase activity of Hsp82p^G313S^ (grey bars) by Sti1p. All reactions contained 2 µM Hsp82p and indicated concentrations of co-chaperones. E. Inhibition of Hch1p-stimulated ATPase activity of wildtype Hsp82p (black bars) and Hsp82p^A587T^ (white bars) by Sti1p. All reactions contained 2 µM Hsp82p and indicated concentrations of co-chaperones. ATPase rates shown in µM ATP hydrolyzed per minute per µM of enzyme (1/min).

Aha1p and Hch1p are two known stimulators of the Hsp90 ATPase activity but the co-chaperones Cpr6p and Tah1p have also been shown to weakly stimulate this activity [21], [52]. Two other co-chaperones, Sba1p and Sti1p, are known inhibitors of this stimulation. Sba1p binds to the *N* terminus of Hsp90 at or near the site where the Aha1p *C* terminus is thought to bind [18], [24], [33]. It is thought that Sba1 binds to the transition state of Hsp90 and slows ATP hydrolysis [53]. In contrast, Sti1p binds to the *C* terminal MEEVD peptide of Hsp90 via one of its TPR domains and enforces an ‘open’ conformation in Hsp90 by preventing N terminal dimerization [17]. We tested Sba1p and Sti1p for the ability to inhibit the Aha1p and Hch1p-stimulated ATPase activity of our Hsp82p mutants. Sba1p was capable of inhibiting the Aha1p-simulated ATPase activity of wildtype Hsp82p, Hsp82p^G313S^ and Hsp82p^A587T^ (Figure 6A) suggesting that Sba1p binding was not affected by either mutation. When we tested Sba1p for the ability to inhibit Hch1p-stimulated ATPase activity of wildtype Hsp82p and Hsp82p^A587T^ (we did not test Hsp82p^G313S^ in this way because its ATPase activity was undetectable in the absence of Aha1p) we found that it was only effective in reactions containing wildtype Hsp82p (Figure 6B). This could mean that Sba1p cannot bind Hsp82p^A587T^ in the presence of Hch1p or that Sba1p can bind to this complex but cannot affect its ATPase activity. We next tested Sti1p for inhibition of Aha1p-stimulated ATPase activity. The Aha1p-stimulated ATPase activity of wildtype Hsp82p, Hsp82p^G313S^, and Hsp82p^A587T^ was efficiently inhibited by Sti1p regardless of the presence or absence of Hch1p (Figure 6C, 6D). Sti1p inhibited the weak Hch1p-mediated stimulation we observed with wildtype Hsp82p and Hsp82p^A587T^ (Figure 6E).

### Interplay between activators and inhibitors of the Hsp82p ATPase activity

**Figure 7:**
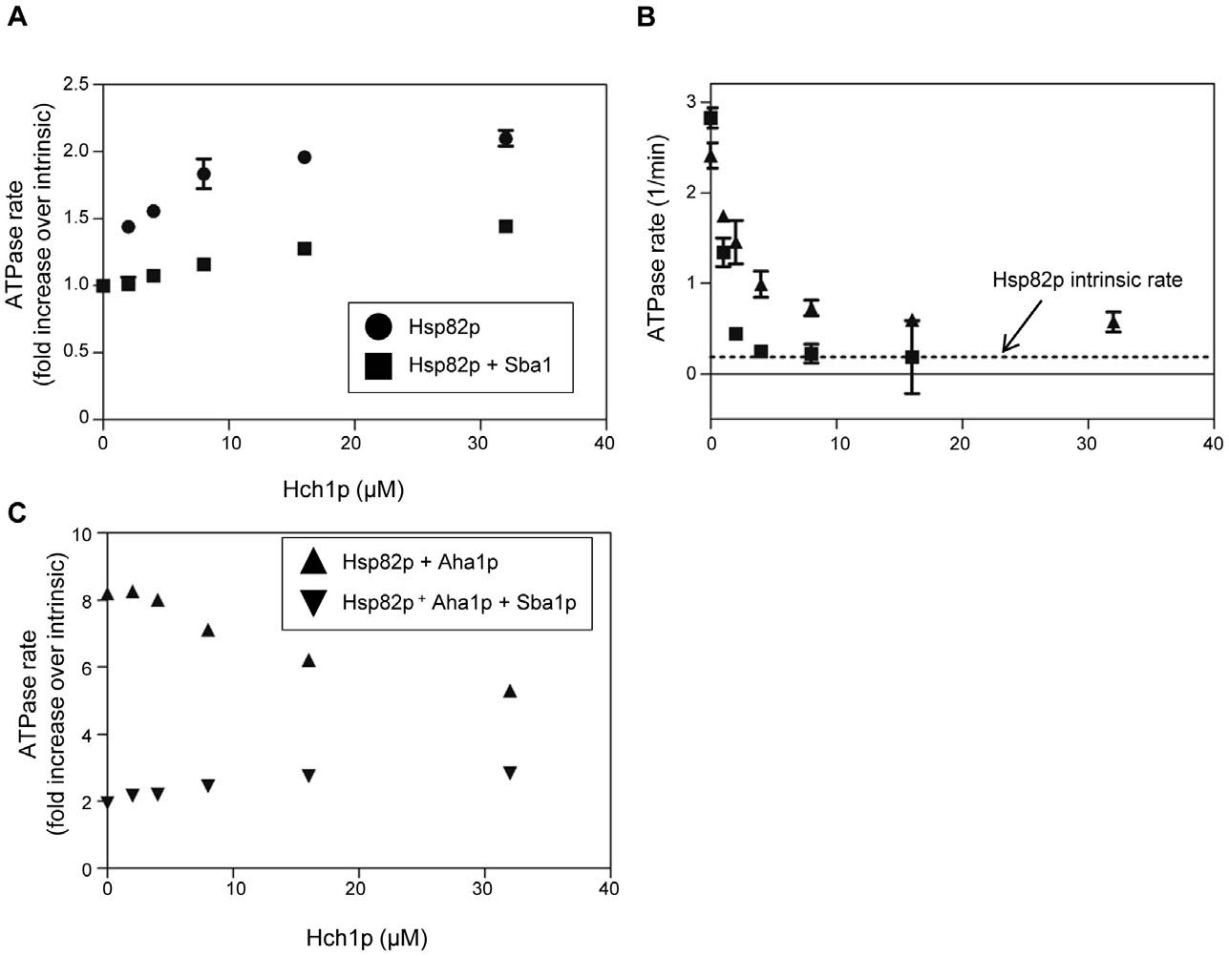
Co-chaperone competition in Hsp82p ATPase reactions. A. Stimulation of the ATPase activity of wildtype Hsp82p (2 µM) by increasing concentrations of Hch1p in the presence (squares) or absence (circles) of Sba1p (8 µM). ATPase rate shown as a fold increase over intrinsic rate of Hsp82p alone. B. Inhibition of Aha1p-stimulated ATPase rate by increasing concentrations of Sba1p (triangles) and Sti1p (squares). Dashed line represents intrinsic Hsp82p ATPase rate. Reactions contain 2 µM Hsp82p, 4 µM Aha1p and indicated concentrations of Sba1p or Sti1p. ATPase rate shown in µM ATP hydrolyzed per minute per µM of enzyme (1/min). C. Effect of increasing concentrations of Hch1p on the Aha1p-stimulated ATPase activity of wildtype Hsp82p (2 µM) in the presence (squares) or absence (circles) of Sba1p (8 µM). ATPase rate shown as a fold increase over intrinsic rate of Hsp82p alone. Reactions contained 2 µM Hsp82p and indicated concentration of Aha1p or Hch1p.

To further characterize the relationship between Aha1p, Hch1p and Sba1p, we added increasing amounts of Hch1p to reactions containing different combinations of Aha1p and Sba1p. We saw a steady increase in ATPase activity when Hch1p was titrated into Hsp82p (Figure 7A). However, when Hch1p was titrated into reactions containing Hsp82p and Sba1p the increase was much more gradual and lower than in the absence of Sba1p (Figure 7A). This is consistent with the observation that increasing amounts of Sba1p, while effective at reducing Aha1p-stimulated (Figure 7B) or Hch1p-stimulated (Figure 6B) ATPase activity, cannot reduce ATPase activity to intrinsic levels) [21]. In contrast and consistent with reports that Sti1p and Aha1p binding to Hsp90 is mutually exclusive, Sti1p can compete Aha1p-stimulated ATPase activity to non-stimulated levels (Figure 7B) [17]. When we titrated Hch1p into Aha1p-stimulated reactions we observed a decrease in ATPase activity suggesting that Hch1p and Aha1p compete for binding to the same site (Figure 7C). Replacing a robust stimulator such as Aha1p with a much weaker one like Hch1p explains this decrease in activity. However, when we added increasing amounts Hch1p to Aha1p-stimulated reactions containing Sba1p we observed a slow increase from the Sba1p-inhibited level (Figure 7C).

### Analysis of Hsp82p complex formation

**Figure 8:**
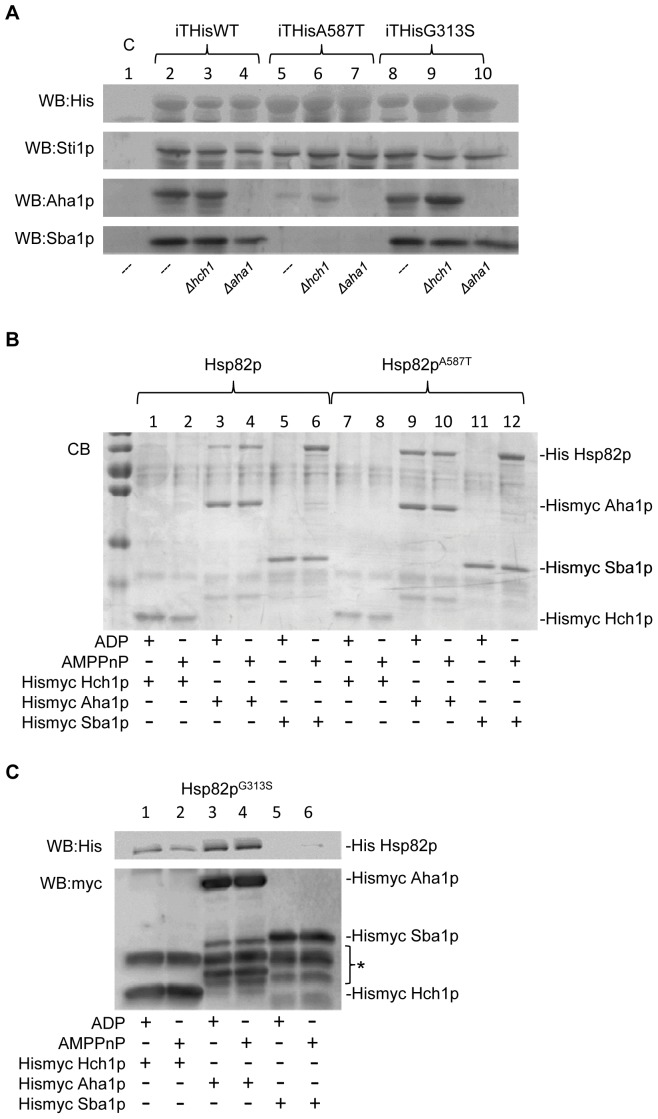
Analysis of complexes formed with Hsp82p, Hsp82p^G313S^ and Hsp82p^A587T^. A. Ni-NTA pulldown of His-tagged Hsp82p from lysates from yeast expressing either untagged wildtype Hsp82p (Lane 1), HisHsp82p (Lanes 2–4), Hsp82p^A587T^ (Lanes 5–7) or Hsp82p^G313S^ (Lanes 8–10). Isolated complexes were analyzed by western blotting with antibodies to the indicated proteins. Genetic background of each strain is indicated beneath each lane. B. 5 µM Hsp82p or Hsp82p^A587T^ was incubated with 5 µM of the indicated His-myc tagged co-chaperone and either 1 mM ADP (odd lanes) or 1 mM AMPPnP (even lanes). Complexes were isolated with beads coupled to anti-myc monoclonal antibody 9E10, run on SDS-PAGE and analyzed by coomassie blue staining (CB). C. 5 µM Hsp82p^G313S^ was incubated with 5 µM of the indicated His-myc tagged co-chaperone and either 1 mM ADP (odd lanes) or 1 mM AMPPnP (even lanes). Complexes were isolated with beads coupled to anti-myc monoclonal antibody 9E10, run on SDS-PAGE and analyzed by western blotting with anti-His tag antibodies and anti-myc tag antibodies. *indicates degradation products and light chain from the beads.

Our yeast growth assays suggest that the A587T and G313S mutations confer a defect to Hsp82p that is exacerbated by Hch1p but not Aha1p - which bind to the same site on the middle domain. To better understand the mechanism of this rescue we isolated His-tagged Hsp82p (wildtype Hsp82p as well as Hsp82p^G313S^ and Hsp82p^A587T^) from yeast lysates using nickel beads and analyzed interacting proteins by western blot. We probed these complexes with antibodies for Aha1p, Sti1p, and Sba1p to see if the deletion of *HCH1* altered the interaction of Hsp82p with any of these co-chaperones *in vivo*. Consistent with previous reports, Hsp82p^A587T^ did not co-precipitate a detectable amount of Sba1p [54] and this interaction was not recovered when *HCH1* was deleted (Figure 8A). Similarly, Aha1p was only weakly recovered with Hsp82p^A587T^ compared to Hsp82p or Hsp82p^G313S^ (Figure 8A). Sti1p was detectable in all our pulldowns suggesting that this interaction was unaffected by our Hsp82p mutations or *HCH1* deletion (Figure 8A). The results of these nickel pulldowns was surprising because our ATPase assays showed that there was no impairment in the Aha1p or Sba1p interaction with Hsp82p^A587T^ (Figures 5 and 6). To confirm this observation, we expressed in *E. coli* and purified Aha1p and Hch1p with a tandem hexa-histidine/myc tag for use in immunoprecipitation experiments with complexes formed *in vitro*. Surprisingly, but consistent with our ATPase data, when we incubated recombinant Hsp82p with myc tagged co-chaperone proteins and immunoprecipitated the complexes that formed using beads coupled to anti-myc antibodies we observed normal interaction between Hsp82p^A587T^ and each of Hch1p, Aha1p and Sba1p. In the case of Hsp82p^G313S^, interaction with these three co-chaperones was very weak and had to be detected by western blotting to the His-tag on Hsp82p^G313S^. Analysis of the complexes that form *in vitro* between these proteins show that Hch1p and Aha1p interact with all of our Hsp82p forms (albeit very weakly with Hsp82p^G313S^) regardless of the presence of ADP or AMPPnP (Figure 8B, C). Consistent with published reports on the Hsp82p-Sba1p interaction, our recombinant Hsp82p, Hsp82p^G313S^ and Hsp82p^A587T^ only bound to Sba1p in the presence of AMPPnP (Figure 8B, C).

## Discussion

Hsp90 is one of the most clinically important chaperone proteins known as it is directly involved in the folding and/or activation of proteins such as the cystic fibrosis transmembrane conductance regulator, oncogenic kinases like Src and B-Raf, and hormone receptors like the estrogen and progesterone receptors [2], [3], [4], [5], [6], [7], [8], [9]. Hsp90 inhibitors have shown some promise as a means of halting pro-proliferative and anti-apoptotic signaling in cancer [55], [56]. More recently, drugs that target Hsp90 co-chaperones like the human homologue of yeast Sti1p have illustrated the importance of understanding how these co-chaperones participate in Hsp90 action [57].

Using yeast as a model to study the Hsp90 system we identified *HCH1* as a key determinant of Hsp90 inhibitor drug sensitivity. We found that *HCH1* deletion restores growth at elevated temperatures in yeast that express either Hsp82p^G313S^ or Hsp82p^A587T^ as the sole source of Hsp90, but also near-wildtype resistance to the Hsp90 inhibitor NVP-AUY922 in these strains. Hch1p overexpression was toxic to yeast expressing Hsp82p^A587T^ (and possibly Hsp82p^G313S^) and resulted in hypersensitivity to NVP-AUY922 in yeast expressing wildtype Hsp82p. Interestingly, deletion of *STI1* makes yeast highly sensitive to Hsp90 inhibitors [47], [58], [59]. Yeast that express Hsp82p^G313S^ are not viable when *STI1* is deleted [38] and overexpression of Sti1p rescues high temperature growth of yeast expressing either Hsp82p^G313S^ or Hsp82p^A587T^ [46]. A simple interpretation of these phenotypes is that the Hsp82p^G313S^ and Hsp82p^A587T^ mutants have a molecular defect that is overcome by Sti1p and made worse by Hch1p. We therefore predicted that Hsp82p^G313S^ or Hsp82p^A587T^ might have lower affinity for Sti1p than wildtype Hsp82p or that Hch1p might antagonize the interaction between Sti1p and either of these Hsp82p mutants. However, our analysis shows that the ATPase activity of both of these mutants was effectively inhibited by Sti1p in a way that was not blocked by Hch1p. Moreover, Sti1p was readily recovered in complex with both of these mutants from yeast lysates and was not increased by *HCH1* deletion.

So what is the molecular impairment of Hsp82p^G313S^ and Hsp82p^A587T^? Owing to the similarity in the growth phenotypes of yeast expressing either of these mutants and how they are altered by co-chaperone deletion and overexpression, we hypothesized that their impairments would be the same. However, we found that the enzymatic properties of Hsp82p^G313S^ and Hsp82p^A587T^ were very different as were the complexes they form *in vivo*. Hsp82p^A587T^ was virtually identical to wildtype in our ATPase assays (except for a very minor difference when Hch1p and Sba1p were used together) while the ATPase activity of Hsp82p^G313S^ was severely impaired. Conversely, Hsp82p^G313S^ was pulled down in complex with the same amount of Sba1p and Aha1p and as wildtype Hsp82p while Hsp82p^A587T^ was in complex with very little of either of these two co-chaperones. Moreover, the recovery of Sba1p or Aha1p with Hsp82p^A587T^ was not improved by *HCH1* deletion. The low recovery of Sba1p with Hsp82p^A587T^ is consistent with a previous study done with the equivalent mutant of the constitutively expressed yeast Hsp90, Hsc82p [54]. Despite the similarity in the growth phenotypes of yeast that express either Hsp82p^G313S^ or Hsp82p^A587T^ our data leads us to conclude that that their molecular impairments may in fact be distinct. In the case of Hsp82p^A587T^, the mutation does not impair Sti1p, Aha1p, Sba1p or Hch1p interaction directly (as indicated by our ATPase experiments with recombinant protein) but could specifically impair client processing. Indeed, in living cells Hsp90 is thought to be in vast excess to individual co-chaperones [60]. Moreover, our data suggests that yeast expressing wildtype Hsp82p can handle much greater substrate loads than required at 30°C (as indicated by their robust growth at elevated temperatures) or when Hsp90 activity is compromised by inhibition (as indicated by yeast growth at high concentrations of NVP-AUY922). Taken together, this suggests that, *in vivo*, co-chaperone proteins may not readily interact with Hsp90 that is not actively engaged with a client protein. Therefore, despite having normal affinity for Hsp82p^A587T^, co-chaperones like Sba1p or Aha1p would not interact with Hsp82p^A587T^ unless client binding and progression to the later stages of the cycle warranted it. While defects in client processing are observed in almost all mutant forms of Hsp90, biochemical comparisons of these mutants are almost always done in the absence of client proteins. Future studies will no doubt benefit from a more client-centric analysis of the cycling and biochemistry of Hsp90 mutants.

The prevailing view is that Hch1p and Aha1p are homologues that differ only in the magnitude of ATPase stimulation of Hsp90. If that is the case, why would the deletion of *HCH1* have such a different effect than the deletion of *AHA1*? Previous reports have shown that the growth of a yeast strain engineered to have very low Hsp90 levels is impaired when both of these genes are deleted suggesting they play redundant roles [22]. Indeed, we were unable to identify a clear difference between Aha1p and Hch1p in their regulation of the wildtype Hsp82p ATPase either on their own or in the context of Sba1p or Sti1p inhibition. Importantly, *HCH1*, but not *AHA1*, deletion resulted in resistance to NVP-AUY922 in yeast expressing wildtype Hsp82p suggesting that Aha1p and Hch1p play distinct roles in the regulation of Hsp82p that may be independent of their ability to stimulate ATPase activity.

In summary, we report here that Hch1p plays an important role in resistance to Hsp90 inhibitor drugs that is distinct from Aha1p. Two Hsp82p mutants (Hsp82p^G313S^ and Hsp82p^A587T^) that depend on Sti1p for their *in vivo* activity [38], [46] are rescued when *HCH1* is deleted. Despite the similarity between the *N* terminus of Aha1p and Hch1p, these two proteins interact with Hsp82p and affect its function in very different ways.
